# Spontaneous regression of bone metastasis from renal cell carcinoma; A case report

**DOI:** 10.1186/1471-2407-6-11

**Published:** 2006-01-13

**Authors:** Takahiro Nakajima, Makoto Suzuki, Soichiro Ando, Tomohiko Iida, Akinobu Araki, Takehiko Fujisawa, Hideki Kimura

**Affiliations:** 1 Division of Thoracic Diseases, Chiba Cancer Center, Japan; 2 Division of Pathology, Chiba Cancer Center, Japan; 3 Department of Thoracic Surgery, Graduate School of Medicine, Chiba University, Japan

## Abstract

**Background:**

Spontaneous regression of metastatic renal cell carcinoma is rarely observed.

**Case presentation:**

Metastatic renal cell carcinoma was identified in a 70-year-old male using computed tomography-guided percutaneous needle biopsy. Two months after the diagnosis, a partial resection of the sternal bone was performed. Pathological examination revealed granulated tissue with bleeding and necrosis but no carcinogenic cells.

**Conclusion:**

We report a pathologically identified case in which a sternal bone metastasis that was noticed two years after radical nephrectomy regressed completely and spontaneously.

## Background

Since the first reported case of spontaneous regression of metastatic renal cell carcinoma in 1928 by Bumpus [1], it has been known that renal cell carcinoma can regress spontaneously, although the frequency of the phenomenon was estimated to be less than 1%, and most cases were observed after a resection of the primary tumor. The site of the regression was most commonly in the lung. The present case of spontaneous regression of metastasis from renal cell carcinoma to the sternum is the first such case to be reported.

## Case presentation

**Figure 1:**
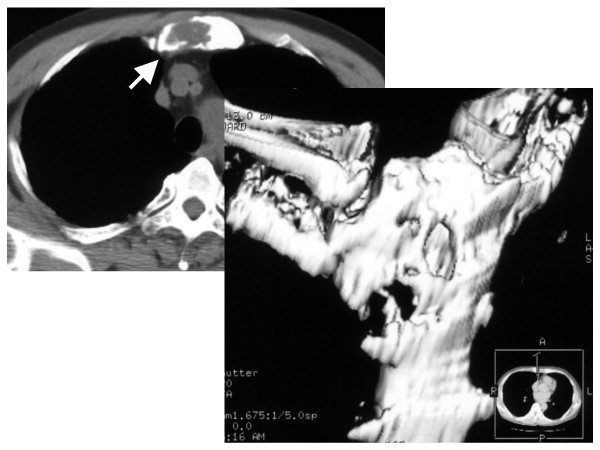
Computed tomographic scan showing a low-density mass measuring 8 × 7 cm that has destroyed part of the cortical bone of the sternum.

**Figure 2:**
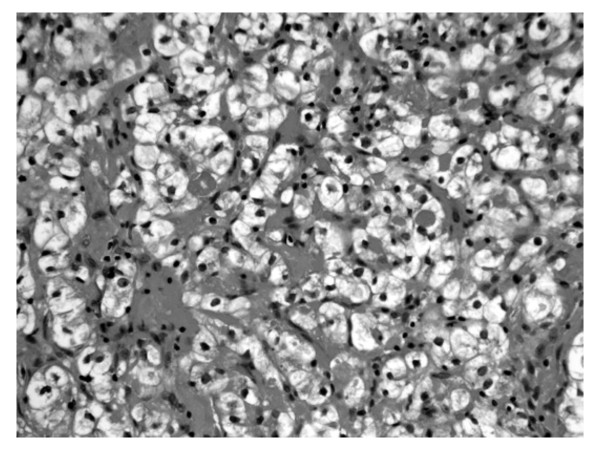
CT-guided percutaneous needle biopsy revealing metastatic renal cell carcinoma. It was composed of clear tumor cells in an alveolar configuration.

**Figure 3:**
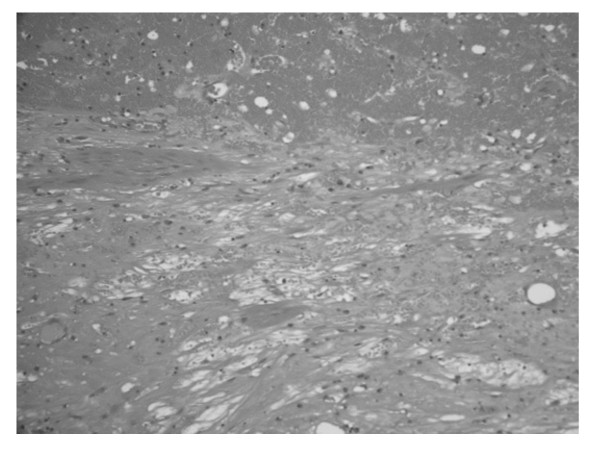
Histological picture of the resected tumor specimen, showing that there were no carcinoma cells although granulation tissue, some bleeding and necrosis were present.

A 70-year-old male was referred to our hospital with anterior chest pain and a precordial mass in September 2004. He had undergone right radical nephrectomy in July 2002, and renal cell carcinoma, T1aN0M0, stage I, had been diagnosed. Medical follow-ups had been conducted routinely without any treatment. In May 2004, after he experienced anterior chest pain, computed tomography (CT) was performed. This revealed in the sternum a low density mass that gradually increased in size during the following three months. In September 2004, there was an 8 × 7 cm mass, and destruction of some of the cortical bone of the manubrium sterni had occurred, so a CT-guided percutaneous needle biopsy was performed. (Fig. 1) The mass was composed of clear tumor cells arranged in an alveolar configuration, and metastatic renal cell carcinoma was diagnosed histologically. (Fig. 2) We performed distant metastasis check up and check his physical function preparing for an operation. No evidence of other distant metastases was seen. We took a chest CT prior to the operation after an admission, the tumor shadow was the same comparing with previous chest CT before the biopsy. The patient underwent a partial resection of the sternal bone with wide margin including whole tumor tissue in November 2004. The postoperative clinical course was uneventful and the patient was discharged 11 days later. Pathological examination revealed only granulation tissue which composed of wide bleeding area and necrosis area with strong infiltration of inflammatory cells especially for fibroblasts. The entire resected tissue specimen was checked carefully, but no carcinoma cells were detected. (Fig. 3) We judged that a complete, spontaneous regression of metastatic renal cell carcinoma had taken place. The patient's medical history was taken in minute detail in relation to any kind of treatment for renal cell carcinoma, but he had received no such treatment and taken no medication, including alternative medicine, nor had he consumed any "health foods" at any time during the course of the disease. He is now receiving interferon therapy and is in good condition without recurrence for eight months after surgery.

## Conclusion

Since Bumpus reported the first case of spontaneous regression of metastatic renal cell carcinoma [1], the incidence of spontaneous regression has been low, and is thought to be less than 1% of all cases [2]. Challis and Stam reviewed the spontaneous regression of cancer from 1900 to 1987, and found that renal cell carcinoma headed the list of histologically diagnosed conditions that underwent spontaneous regression [3].

The sites of regression observed in the reported cases included the brain, bone and liver, but the highest incidence was in the lung [4,5]. In most cases, the tumor regressions were observed following treatments of the primary tumors such as surgical removal, radiotherapy, embolization and radio frequency ablation [4,6]. It is reported that complete regression is even rarer than partial regression. The duration of tumor regression is variable and recrudescence is sometimes observed [5]. The mechanism of spontaneous regression is still unclear, although immunologic effects have been discussed.

In the present case, early-stage renal cell carcinoma was diagnosed. The patient underwent no tumor-specific treatment for about two years after the nephrectomy. The size of the sternal tumor gradually increased until the percutaneous needle biopsy was performed, but it regressed completely within a mere two months. During these two months, the patient had no chemotherapy or immunotherapy, or any kind of interventional treatment. Complete regression of a metastatic tumor is quite rare especially two years after the resection of the primary tumor. One of the possible explanations of this phenomenon may in this case have been induced by the needle biopsy performed in order to diagnose the metastasis; or bleeding caused by the biopsy may have triggered a chain reaction of host immune responses. The patient is now receiving interferon therapy in our outpatient clinic on individual basis rather than supported by literature data to prevent recurrence of the tumor [7-9]. This is the first reported case of spontaneous regression occurring in a sternal bone metastasis from renal cell carcinoma.

## Competing interests

The author(s) declare that they have no competing interests.

## Authors' contributions

TN conceived of the case, participated in the sequence alignment, drafted and revised the manuscript before and following the peer review. MS was the main operator in charge of the case and gave the ideas of this manuscript. SA, TI and TF made substantial contributions to the conception and interpretation of clinical data and case-related studies. AA was the pathologist. HK, the corresponding author, was the operator in charge of this case and made clinical decision, the ideas of this manuscript. All authors read and approved the final manuscript.

## Pre-publication history

The pre-publication history for this paper can be accessed here:
